# A Further Finite Element Stress Analysis of Angled Abutments for an Implant Placed in the Anterior Maxilla

**DOI:** 10.1155/2015/560645

**Published:** 2015-02-23

**Authors:** Dong Wu, Kebin Tian, Jiang Chen, Hua Jin, Wenxiu Huang, Yuyu Liu

**Affiliations:** ^1^School of Stomatology, Fujian Medical University, Fuzhou, Fujian 350000, China; ^2^Department of Oral Implantology, Affiliated Stomatological Hospital of Fujian Medical University, Fuzhou, Fujian 350002, China; ^3^Key Laboratory of Oral Medicine, Guangzhou Institute of Oral Disease, Stomatology Hospital of Guangzhou Medical University, Guangzhou 510140, China; ^4^Department of Stomatology, Hospital of Guangdong Crop Chinese People's Armed Police Forces, Guangzhou, Guangdong 510507, China

## Abstract

To systematically measure and compare the stress distribution on the bone around an implant in the anterior maxilla using angled abutments by means of finite element analysis, three-dimensional finite element simplified patient-specific models and simplified models were created and analyzed. Systematically varied angled abutments were simulated, with angulation ranging from 0° to 60°. The materials in the current study were assumed to be homogenous, linearly elastic, and isotropic. Force of 100 N was applied to the central node on the top surface of the abutments to simulate the occlusal force. To simulate axial and oblique loading, the angle of loading was 0°, 15°, and 20° to the long axis of implant, respectively. There was the strong resemblance between the response curves for simplified patient-specific models and simplified models. Response curves under oblique loading were similar in both models. With abutments angulation increased, maximum von Mises stress firstly decreased to minimum point and then gradually increased to higher level. From a biomechanical point of view, favorable peri-implant stress levels could be induced by angled abutments under oblique loading if suitable angulation of abutments was selected.

## 1. Introduction

In the majority of cases for dental implants in the anterior maxilla, the use of angled abutments has become an increasingly common practice because of patients' and clinicians' expectations [1–4]. The need to change the abutments angle has been recognized, as a result of difference in angle between the bone available for implant placement and the long axis of the planned restoration [5]. The clinical success rates of angled abutments have mostly been satisfactory. Moreover, there are a number of advantages of the usage of angled abutments [3–7]: facilitating paralleling nonaligned implants; aiding the clinicians in avoiding anatomical structures when placing the implants; reducing treatment time, fees, and the need to perform guided bone regeneration procedures.

The influence of angled abutments on stress is a matter of debate [8–10]. It is widely accepted that increased stress on implants and bone has been associated with the use of angled abutments [7, 11, 12]. However, a few studies [8, 13, 14] showed that angled abutments would favor a better distribution of stress within peri-implant bone. Abutments angulation is an important biomechanical variables that need further scientific evaluation [15, 16]. The influence of abutments angulation on stress with peri-implant bone is related to a variety of factors such as loading condition, quality and quantity of jawbone, implant geometry, and surface structure. The wide variety of results from finite element analysis occurred because of different assumptions (Table 1) to be made concerning these biological factors, such as conditions between materials and components, jawbone model (patient-specific models and simplified models), and loading angle (axial and oblique loading). Previous studies [8, 11, 13] compared angled abutments (0°, 15°, 20°, 25°) with straight abutments directly when assessing the influence of angled abutments on stress within peri-implant bone, but it is unclear how a systematic change in the abutments angulation affects the magnitude and pattern of stress in the implant and jawbone. A thorough investigation of stress in surrounding bone of implants is of vital importance to understand the biomechanical behavior of angled abutments. The aim of this study was systematically to measure and compare stress within peri-implant bone using different abutments in which angulation was ranged from 0° to 60° in different jawbone models (simplified patient-specific models and simplified models) by means of finite element analysis and gaining systematical insight into the influence of angled abutments on stress distribution on the bone surrounding the implant in the anterior maxilla.

## 2. Methods 

**Figure 1:**
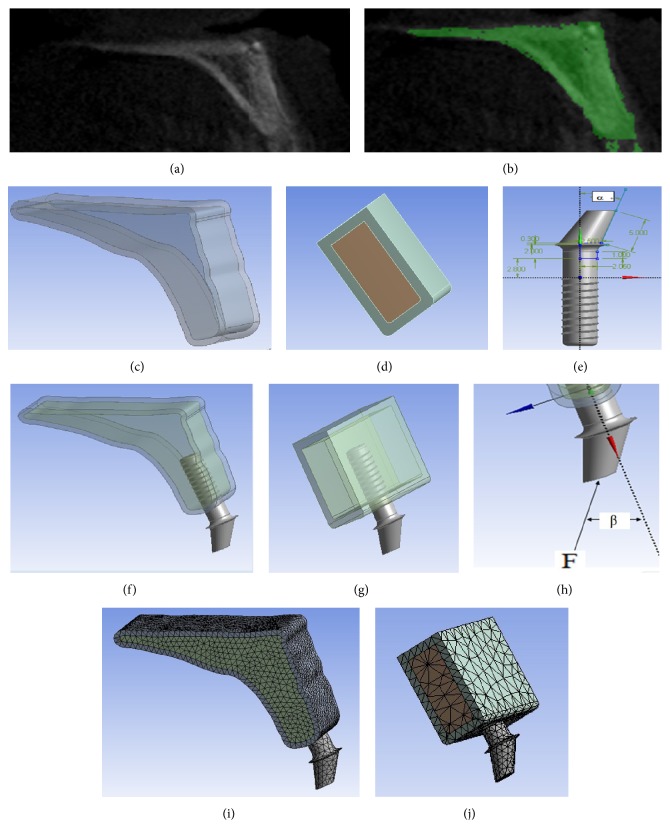
Simplified patient-specific models: A cone-beam computerized tomography scan projection of a maxillary central incisor region was obtained (a), the outline of the image was manually converted (b). Simplified patient-specific models were created by extruding the simplified cross-sectional image (c). Simplified models were used in this study (d). The geometry of the implant-abutment complex was developed based on the models described in the previous study;  *α* was abutment angulation (e). All models were combined by Boolean operations (f, g). (h)* F*: occlusal force;  *β* (loading angle) = 0°, 15°, and 20°. (i, j) Meshed implants and bone.

For the present study, two different three-dimensional finite element models are as follows. Simplified patient-specific models and simplified models were created and analyzed using ANSYS 9.0 software (ANSYS, Canonsburg, PA). Simplified patient-specific models are as follows. A cone-beam computerized tomography scan projection of a maxillary central incisor region (Figure 1(a)) was obtained from the Department of Oral and Maxillofacial Radiology, Affiliated Stomatological Hospital of Fujian Medical University. To simplify analysis, the outline of the image was manually converted and palatine segment was cut off (Figure 1(b)). The simplified cross-sectional image was then extruded to create an anterior maxilla segment. The dimensions of the anterior maxilla segment are shown in Figure 1(c). The overall dimensions of the bone model were 20 mm in vertical height, 20 mm in mesiodistal length, and 9 mm in labiopalatal width at the ridge crest. The average thickness of the cortical bone in the crestal region was 1.5 mm. The mesial and distal planes were not covered by cortical bone. The simplified models (Figure 1(d)) were approximately 9 mm in width buccolingually and 20 mm in height coronoapically and 20 mm in length mesiodistally. The simplified models consisted of two layers: a cortical layer and a cancellous layer. The cortical bone was modeled as a 1.5 mm layer on the facial, lingual, and occlusal aspects of the bone wedge. The geometry of the implant-abutments complex (Figure 1(e)) was developed based on the models described in the previous study [13]. A modification was made such that the abutments angulation was a variable factor. The angulations of abutments were adopted from the commonly used angled abutments available from the relevant literature [1, 2, 5, 17]. Systematically varied angled abutments were simulated, with angulation ranging from 0° to 60°. All models were combined by Boolean operations (Figures 1(f) and 1(g)). All of the materials in the current study were assumed to be homogenous, linearly elastic and isotropic to simplify computation processes. The mechanical properties (Table 2), boundary conditions, and the nature of loading were obtained from relevant studies [2, 13]. Occlusal forces are typically 100 N under normal biting, with higher forces occurring in patients suffering from bruxism or parafunction. Force of 100 N was applied to the central node on the top surface of the abutments to simulate the occlusal force [13, 18]. Axial and oblique loading were applied to each model. The angle of oblique loading was 15° and 20° to the long axis of implant (Figure 1(h)), because most occlusal loads applied to anterior teeth were at an angled to the long axis the implant [12]. Direction of axial loading was parallel to the long axis of implant; the angle of loading was 0° to the long axis of implant. The interface between the cortical and cancellous bone layers and between the implant and each of the bone layers was assumed to be properly bonded to correspond with good osseointegration. The lower surface of the model and the medial and distal planes of bone were completely constrained [17]. The numerical models were meshed with 1.0 mm of element sizing (Figures 1(i) and 1(j)). For angled abutments, dental implants, cortical bone, and trabecular bone, a 10-node solid element of SOLID 187 was used. Meshed simplified patient-specific models showed a number of nodes ranging 87,236 to 87,844 and number of elements ranging from 53,069 to 53866 (Figure 1(i)). Simplified models were composed of 39,000 nodes and 16,000 elements with a small difference in various models (Figure 1(j)). The maximum von Mises stress for cortical and cancellous bone was recorded. Abutments angulation was set as the input variables. The maximum von Mises stress was set as output variables to evaluate the effect of different abutments angulation on the jaw bone and implant. The response curves of input variables to output variables were constructed.

## 3. Results

**Figure 2:**
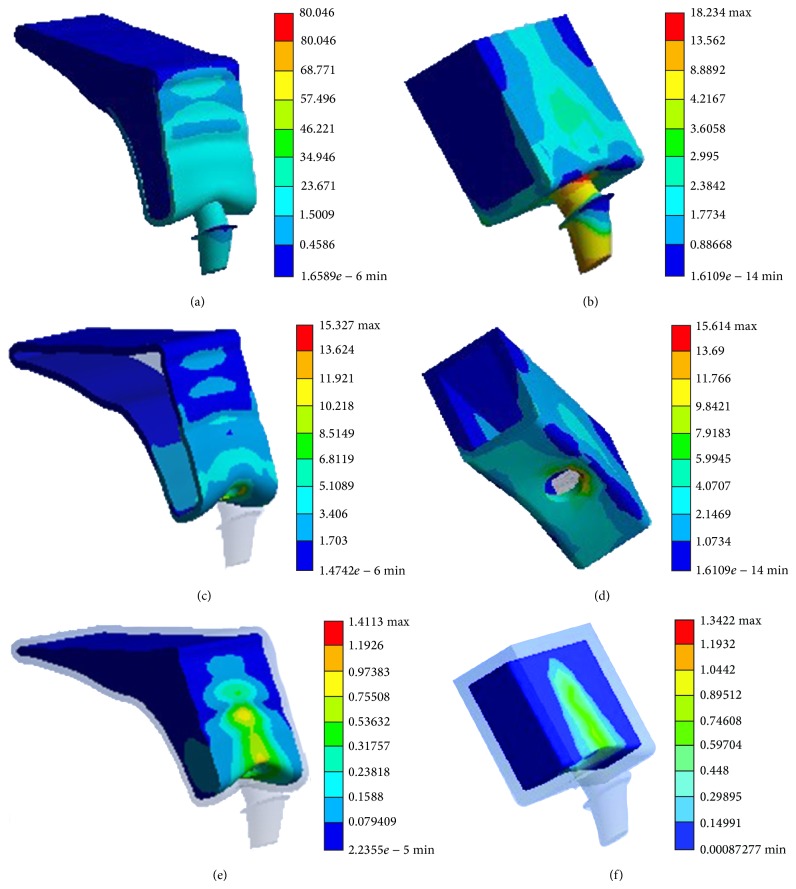
The general patterns for stress distribution were similar for simplified patient-specific models (a) and simplified models (b). Distribution of maximum von Mises stress (MPa) in cortical bone (c, d) and cancellous bone (e, f) for simplified patient-specific models and simplified models, respectively. Blue to red colors represent stress values from lower to higher.

**Figure 3:**
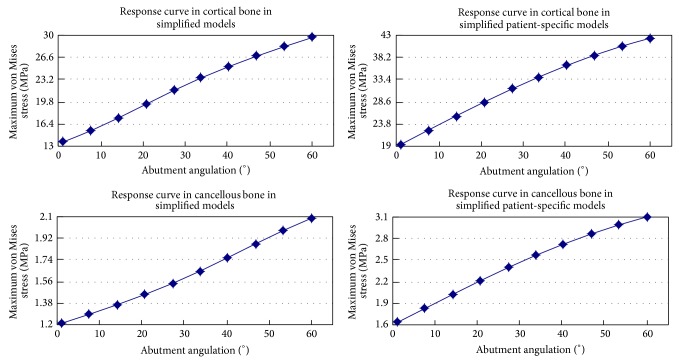
Response curve of abutment angulation to maximum von Mises stress in cortical bone and cancellous bone under axial loading in simplified patient-specific models and simplified models.

**Figure 4:**
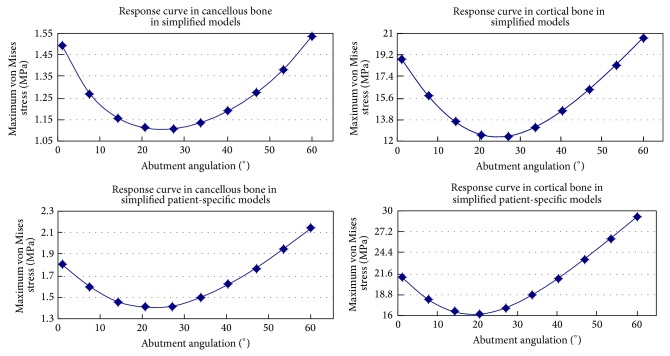
Response curve of abutment angulation to maximum von Mises stress in cortical bone and cancellous bone under 15° oblique loading in simplified patient-specific models and simplified models.

**Figure 5:**
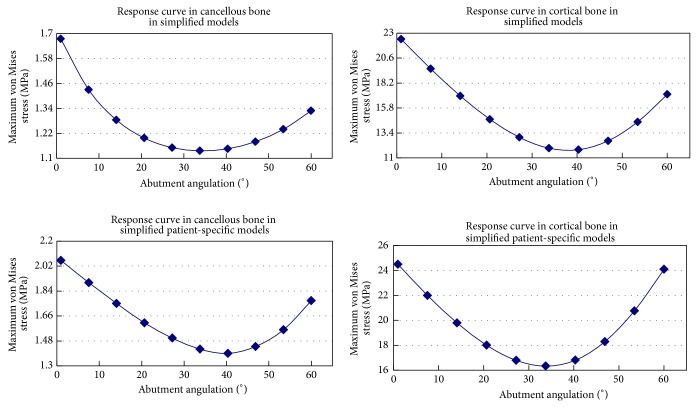
Response curve of abutment angulation to maximum von Mises stress in cortical bone and cancellous bone under 20° oblique loading in simplified patient-specific models and simplified models.

Numerical and graphic results were generated for maximum von Mises stress. According to different jawbone models, two samples were modeled in this study. The general patterns for stress distribution (Figures 2(a) and 2(b)) were similar for all models. High stress values were located at cervical cortical bone regions adjacent to implants (Figures 2(c) and 2(d)). Relatively low stress values were identified in cancellous bone regions (Figures 2(e) and 2(f)) due to the lower elastic property of this type of bone compared to cortical bone. Variation of the maximum von Mises stress and response curves of abutments angulation versus maximum von Mises stress in simplified patient-specific models and simplified models under axial loading angle are shown in Figure 3, respectively. The response curves show the strong resemblance between simplified patient-specific models and simplified models. The magnitude of maximum von Mises stress in cortical and cancellous bone increased with an increase in the abutments angulation. In simplified models when the angulation of angled abutments was changed from 0° to 60° maximum von Mises stress increased by 75% and 117%, from 1.2 MPa to 2.1 MPa and 13.7 MPa to 29.6 MPa in cancellous and cortical bone, respectively. In simplified patient-specific models when the angulation of angled abutments was changed from 0° to 60° maximum von Mises stress increased by 94% and 116%, from 1.6 MPa to 3.1 MPa and 19.5 MPa to 42.2 MPa in cancellous and cortical bone, respectively. Variation of the maximum von Mises stress and response curves of abutments angulation versus maximum von Mises stress in simplified patient-specific models and simplified models under oblique loading (15° and 20°) are shown in Figures 4 and 5, respectively. Response curves of abutments angulation to maximum von Mises stress basically were also similar in both models. With abutments angulation increasing, maximum von Mises stress firstly decreased to minimum point and then gradually increased to higher level. In simplified models under oblique loading (15°) maximum von Mises stress in cancellous and cortical bone firstly reduced by 26% and 34%, from 1.5 MPa to 1.1 MPa and from 18.9 MPa to 12.5 MPa, and then increased to 1.5 MPa and 20.1 MPa, respectively. In simplified patient-specific models under oblique loading (15°) maximum von Mises stress in cancellous and cortical bone firstly reduced by 22% and 23%, from 1.8 MPa to 1.4 MPa and from 21.1 MPa to 16.2 MPa, and then increased to 2.1 MPa and 29.1 MPa, respectively. In simplified models under oblique loading (20°) maximum von Mises stress in cancellous and cortical bone firstly reduced by 31% and 47%, from 1.6 MPa to 1.1 MPa and from 22.4 MPa to 11.8 MPa, and then increased to 1.3 MPa and 17.1 MPa, respectively. In simplified patient-specific models under oblique loading (20°) maximum von Mises stress in cancellous and cortical bone firstly reduced by 33% and 33%, from 2.1 MPa to 1.4 MPa and from 24.5 MPa to 16.3 MPa, and then increased to 1.7 MPa and 24.1 MPa, respectively.

## 4. Discussion

A few of the studies have investigated the angled abutments in anterior maxilla by means of finite element analysis. Clelland et al. [11] used a three-dimensional finite element analysis model of maxilla and confirmed that stress became larger as abutments angulation increased. They also noted that peak compressive stress for the 20° angled abutments was slightly above this physiological zone. Nothdurft et al. [8] in a two-dimensional finite element analysis study predicted a 15% higher maximum bone strain for the straight compared with the angled abutments. But most of the strain produced on the cancellous and cortical bone was within the range that has been reported to increase bone mass and mineralization. Kao et al. [12] investigated the influence of abutments angulation upon the micromotion level and stress distribution pattern for an immediate loading single implant placed in anterior maxillary region. The micromotion values were 19% and 34% greater than those of the straight abutments for abutments angles of 15° and 20°. Compared to straight abutments, the 25° abutments result in increased maximum von Mises stresses to a level of 18%. Tian et al. [13] designed four simplified models to simulate clinical scenarios in which those implants were placed in an ideal axial position or at an angled position. Their study showed that angled abutments could result in decreased stress on the supporting bone of implant system under certain conditions. This result suggests that angled abutments may be a suitable restorative option when implants are not placed in the ideal axial position. Bahuguna et al. [19] modelled the frontal region of maxilla with a cortical layer containing an inner cancellous core. The different abutments angulations used were 0°, 10°, 15°, and 20°. These were then subjected to axial and oblique loadings. The study showed that as the abutments angulation changes from 0° to 20° both compressive and tensile stresses increased, but it is within the tolerance limit of the bone. Most of studies agreed with that angled abutments result in increased stress on the implants and adjacent bone, and these increased stresses usually are within physiological tolerances [11, 19]. Most of studies have predicted that use of angled abutments on implant results in a greater amount of stress in bone. There are contradictory findings concerning the effect of angled abutments. Nothdurft et al. [8] and Tian et al. [13] specifically appraised the effect of abutments angulation on bone surrounding implants, because different assumptions (Table 1) were made concerning these biological factors, such as conditions between materials and components, jawbone models, and loading condition. A systematic investigation of stress in surrounding bone of implant is needed to fully understand the biomechanical behavior of angled abutments. So systematically varied angled abutments was simulated, with angulation ranging from 0° to 60°, simplified patient-specific models constructed from computed tomography images and simplified models were compared, and axial and oblique loading were investigated in this study. Esthetic demands and nonparallel situations between the axial direction of the suprastructure and the implant require angled abutments [17]. Most implant manufacturers offer at least one prefabricated abutments and have the facility for fabricating customized abutments [1, 2]. The majority (90.2%) of angled abutments used ranged between 5° and 30°. A small number (9.8%) of 35°, 40°, and 45° abutments were also used [5]. The clinical report described the prosthetic treatment of severely malpositioned implants; 55° abutments were used to ensure no tissue impingement [17]. Previous study suggests a need to evaluated greater abutments angulations. So greater abutments angulation (from 0° to 60°) was evaluated in this study.

Two kinds of response curves of abutments angulation versus maximum von Mises stress were shown in both models. The first kind of response curves in Figure 3 was consistent with most of previous studies concerning the effect of angled abutments: the magnitude of maximum von Mises stress within peri-implant bone increased with an increase in the abutments angulation. The second kind of response curves in Figures 4 and 5 showed that with abutments angulation increasing, maximum von Mises stress firstly decreased to minimum point and then gradually increased to higher level. It may support that angled abutments would favor a better distribution of stress within peri-implant bone under certain condition. The reason for the difference was different loading conditions in this study: axial loading for the first kind of response curves and oblique loading for the second one. The first kind of response curves confirmed the conclusion which was widely accepted that this was an increase in magnitude of stress as the abutments angulation increased. The debate about the influence of angled abutments maybe related to the second kind of response curves. For example, response curve in cortical bone under 15° oblique loading in simplified models is shown in Figure 3. When the abutments were straight abutments (angulation = 0°), maximum von Mises stress was 18.8 Mpa; it decreased to 12.5 Mpa with an increase in abutments angulation to 27° and then increased to 20.6 Mpa as the abutments angulation reached 60°. When the angulation of abutments changed from 0° to 27°, maximum von Mises stress decreased as the abutments angulation increased. When the angulation of abutments changed from 27° to 60°, maximum von Mises stress increased as the abutments angulation increased. Response curves showed the strong resemblance in simplified patient-specific models and simplified models under oblique loading (15° and 20°). The loading condition was a significant factor influencing the stress in peri-implant bone [10, 20]. Oblique loading was unfavorable for stress distribution in both bone and implants [21]. Under oblique loading, the stress was distributed asymmetrically in bone, with the maximum value located on the opposite of the loading [20]. When angled abutments were used, the stress was also distributed asymmetrically in bone, with the maximum value located on the site of the direction of abutments [11, 12, 22]. When the direction of loading was opposite to the direction of angled abutments, angled abutments resulted in decreased stress on surrounding bone of implants in previous studies [8, 13, 14]. So it was reasonable to conclude that angled abutments could result in beneficial stress on surrounding bone of implants under oblique loading if suitable angled abutments were used. This result could be used to explain the wide variety of study from various researchers: the magnitude of stress within peri-implant bone increased with an increase in the abutments angulation under axial loading; it was possible that the magnitude of stress within peri-implant bone increased or decreased with an increase in the abutments angulation under oblique loading.

Prefabricated angled abutments are uniform, standardized, and easy to use and have an excellent fit. However, if the position or angulation of the fixture is not appropriate, it is difficult to use prefabricated abutments. Such difficulties may be overcome by customized abutments with computer-assisted design/computer-assisted manufacturing systems [23]. However, the results in this study showed that the optimized abutments angulation could reduce maximum von Mises stress by 22–47%. Maximum von Mises stress firstly decreased to minimum point and then increased to higher level in response curve under 15° and 20° loading angle. From a biomechanical point of view, when the stress reached minimum point the corresponded abutments angulation may be the optimal angulation of angled abutments. Thus, except advantages of accuracy fit and esthetic emergence profile, another advantage of customized angled abutments that may be added is a more favorable distribution of stress [24].

Although absolute values differed, response curve showed similar tendencies between simplified patient-specific models and simplified models, and thus, when absolute values are not of interest, the simplified models could be effectively used in qualitative finite element analysis of angled abutments in the maxilla. Using simplified models can considerably enhance cost-performance and efficiency of comparative stress analyses. Patient-specific models constructed from computed tomography images may simulate clinical situations much more accurately [25, 26]. Thus, if absolute stress values would be the purpose of further finite element analysis, the patient-specific models could be a more appropriate simulation of maxillary bone. But the simplified models offer a more expedite and cost-efficient way to biomechanically investigate and compare angled abutments shapes and angulation.

Dental implant unlike natural tooth supported by periodontal ligaments to help buffer occlusal forces can be more sensitive to occlusal loading, in which severe stress may lead to damage on the bony tissues around implant. On the other hand, an inadequate mechanical stimulus may reduce bone engagement and lead to disuse resorption in the bone. It is difficult for clinicians to define a stress threshold range that would induce disuse and overloading bone resorption around an implant [7, 27]. Just as most of finite element studies, in this study the assumption was made that reducing peak stress is an important issue in promoting and maintaining osseointegration [21, 28, 29] and only maximum von Mises stress was considered [30]. The structures in the model were all assumed to be homogenous and isotropic. All interfaces between the materials were assumed to be bounded. The prosthesis of dental implant was not modeled, and its effect on stress was also not taken into account. Therefore, the present models cannot provide absolute values of stress in the bone around implant with angled abutments and thus may not be quantitatively validated by a clinical study. However, for a comparative study, such simplifications are considered to be reasonable [31, 32]. These data may provide a valuable reference for clinical practices. Further research should be conducted to confirm these numerical results in experimental and clinical studies. And the cost to build a customized implant versus a commercial one would be discussed in future research [33].

## 5. Conclusions 

Within the limitations of this study, the following conclusions were drawn.

1. The magnitude of stress within peri-implant bone increased with an increase in the abutments angulation under axial loading; it was possible that the magnitude of stress within peri-implant bone increased or decreased with an increase in the abutments angulation under oblique loading.
2. From a biomechanical point of view, favorable peri-implant stress levels may be induced by angled abutments under oblique loading if suitable angulation of abutments was selected.

## Figures and Tables

**Table 1:** Different assumptions concerning jawbone model, loading condition in previous and present studies.

	Jawbone models	Angulation of abutments	Loading condition
Clelland et al. [11]	Models constructed from computed tomography images	0°, 15°, and 20°	178 N applied along the long axis of abutment
Saab et al. [9]	Models constructed from computed tomography images	20°	178 N oblique loading
Kao et al. [12]	Simplified models	0°, 15°, and 25°	89 N oblique loading
Tian et al. [13]	Simplified models	0° and 20°	100 N axial and oblique loading
Bahuguna et al. [19]	Simplified models	0°, 10°, 15°, and 20°	100 N, 125 N, 150 N, 175 N, and 200 N axial loading; 50 N oblique loading.
Present study	Simplified patient-specific models and simplified models	From 0° to 60°	100 N axial and oblique loading

**Table 2:** Mechanical properties of materials.

	Young's modulus [MPa]	Poisson's ratio
Cortical bone [2, 13]	13,700	0.30
Cancellous bone [2, 13]	1,370	0.30
Titanium alloy [13]	110,000	0.30
